# Influence of neoadjuvant chemotherapy on prognosis of patients with synovial sarcoma

**DOI:** 10.1186/s12957-017-1165-9

**Published:** 2017-05-11

**Authors:** Yanan Wu, Wenzhi Bi, Gang Han, Jinpeng Jia, Meng Xu

**Affiliations:** Department of Bone Tumor, The General Hospital of the People’s Liberation Army, Beijing, 100853 China

**Keywords:** Synovial sarcoma, Neoadjuvant chemotherapy, Overall survival, Freedom from distant metastasis

## Abstract

**Background:**

This study aimed to explore the clinical efficacy of neoadjuvant chemotherapy combined with surgery in primary synovial sarcoma of the limbs and trunk through retrospective analysis of patients with primary synovial sarcoma of the limbs and trunk treated by this treatment in our hospital.

**Methods:**

A total of 89 patients diagnosed with synovial sarcoma were enrolled in this study between January 2005 and December 2011 in PLA General Hospital. Most of the patients received neoadjuvant chemotherapy combined with operative treatment (84.3%), 10.1% of them received adjuvant chemotherapy combined with operative treatment, and only 5.6% received merely operative treatment. The influence on the prognosis of patients with synovial sarcoma was analyzed by the statistics overall survival (OS), progression-free survival (PFS), local control (LC), and freedom from distant metastasis (FFDM).

**Results:**

The median follow-up time was 68.6 months. The 5-year OS, 5-year PFS, 5-year LC, and 5-year FFDM of the patients were 80.2, 60.5, 78.8, and 80.8%, respectively. The OS of the patients with a tumor size >5 cm was lower (91.4 vs 73.1%, *P* < 0.05). Besides, the OS and FFDM of neoadjuvant chemotherapy were better than those of adjuvant chemotherapy (84.5 vs 55.6%, *P* = 0.015, and 83.8 vs 55.6%, *P* = 0.028, respectively). However, there was no significant difference in the LC and PFS.

**Conclusions:**

Neoadjuvant chemotherapy was beneficial for patients with synovial sarcoma, and it could improve survival time and control distant metastasis. Tumor size was an important factor influencing patients’ prognosis.

## Background

Synovial sarcoma is a high-grade soft tissue sarcoma with poor prognosis and accounts for 5~10% of soft tissue sarcomas [1]. It commonly occurs in young people; the age of onset is usually 15 to 40 years old. Synovial sarcoma is not tumor derived from synovial tissues, but it was named as such because it is frequently seen in soft tissues around the joints, which were mistaken to be derived from synovial tissues. However, it has been proved that synovial sarcoma could occur in multiple sites and organs. The origin of synovial sarcoma is not very clear, and nervous tissue, muscular tissue, and mesenchymal stem cells are likely to be its source [2]. Pathologically, synovial sarcoma is divided into unipolar type, bipolar type, and undifferentiated type. It possesses a constant chromosome translocation, commonly presenting a t(X;18)(p11;q11) balanced translocation. This translocation includes gene fusion between the SYT gene in chromosome 18 and the SSX1 or SSX2 gene in chromosome X and occasionally gene fusion with SSX4 [3]. Synovial sarcoma can occur in all parts of the body, but 80% of it is in the extremities [4]. It also easily metastasizes to organs, 70% to the lung and 10~20% to the bone. Whether synovial sarcoma has a propensity to metastasize to regional lymph nodes still remains a matter of debate [5, 6]. Weingrad and Rosenberg reported that lymphatic metastasis accounted for 17% of synovial sarcoma metastases [7].

In a previous study, it has been reported that the influencing factors for the prognosis of patients with synovial sarcoma included age, tumor size, tumor site, tumor staging, tumor pathological pattern, treatment, and surgical resection margin, which are still controversial. In order to further analyze and obtain the influencing risk factors for the prognosis of the disease, 89 patients with synovial sarcoma were enrolled in this 9-year single-center study. According to whether they were receiving neoadjuvant chemotherapy, the patients were divided into two groups. Through a comparative analysis of patient situation and prognosis in the two groups, the influence of neoadjuvant chemotherapy on the patients’ prognosis was demonstrated.

## Methods

### Patients

A total of 89 patients diagnosed with synovial sarcoma were enrolled in this study between January 2005 and December 2011 in PLA General Hospital, and the last follow-up time was December 2014. The inclusion criterion is confirmed pathological diagnosis by an authorized pathologist in our hospital through tumor histological characteristics of the biopsy specimen and immunohistochemistry. The exclusion criteria are as follows: (1) patients diagnosed with synovial sarcoma but with distant metastases, (2) patients with other tumor history during or before the diagnosis of synovial sarcoma, (3) patients who received radiotherapy after being diagnosed with synovial sarcoma.

Tumor staging was done based on the patients’ medical history, physical examination, chest CT examination, X-ray, and MRI of local primary tumors. The relevant medical records were identified by the Ethics Committee of PLA General Hospital, and all the patients had signed the informed consent form.

### Treatment

The main treatment for soft tissue tumor was neoadjuvant chemotherapy and extended resection of local tumor. When the tumor had invaded important nerves and blood vessels, and extended resection could not guarantee the patient’s safety after resection, amputation was done. The patients receiving neoadjuvant chemotherapy received chemotherapy for approximately one to three treatment courses before surgery, approximately one to six treatment courses after surgery, and one repeat course every 3~4 weeks. The specific condition of the chemotherapy course was made according to the tolerance degree of the patients on chemotherapy drugs, response after tumor chemotherapy, and family economic status. MAID (mesna, adriamycin, ifosfamide, and dacarbazine) protocols were used to treat the patients. The dose, administration method, and administration time of the chemotherapy drugs were based on the protocol shown in Table 1. As for the patients receiving neoadjuvant chemotherapy, after the last chemotherapy before surgery, we performed a routine MRI test on the tumor site and made a surgery plan according to the MRI features. Moreover, we still used MAID protocols to the patients with adjuvant chemotherapy for approximately one to six treatment courses after surgery.

**Table 1:** Different types of chemotherapy drugs, dosage, and administration method and time

Chemotherapy drug	Dosage	Administration method	Administration time
Ifosfamide	2 g/m^2^/day	Intravenous drip	D1–D5
Dacarbazine	300 mg/m^2^/day	Intravenous drip	D1–D5
Adriamycin	40 mg/m^2^	Intravenous drip	D5

### Assessment criteria for the results

Based on the survival state and clinical manifestation in the final follow-up, overall survival (OS), progression-free survival (PFS), local control (LC), and freedom from distant metastasis (FFDM) were analyzed. The starting time of treatment was defined as the chemotherapy or surgery time for the first time in our hospital. The starting and the ending time of related indexes were from the treatment to the occurrence of related events or the final follow-up. The ending related events for related indexes were as follows: for OS, death caused by any reason; for PFS, occurrence of relapse or metastasis; for LC, occurrence of local recurrence; for FFDM, occurrence of distant metastasis.

### Statistical analysis

The data was analyzed by SPSS 19.0 (IBM, Chicago, USA). Measurement data with normal distribution were presented as mean ± SD, and counted data were presented as percentage (%). Comparisons between groups of measurement data with normal distribution were analyzed by the *t* test, and counted data were analyzed by the *χ* ^2^ test. Survival analysis was done by the Kaplan-Meier survival curve and log-rank test. Univariate and multivariate analyses were conducted by the Cox regression model. All the statistical tests were bilateral.

## Results

### Baseline information of patients

There were 46 males and 43 females with an average age of 32.5 years (aged 10~70) in this study. Besides, 30 patients were less than 25 years old at the time of initial diagnosis. The average tumor size was 6.5 cm (3~17 cm). Thirty-five patients had tumor sizes smaller than 5 cm. The distributions of the tumor site were as follows: lower limb 52 cases (58.4%), upper limb 23 cases (25.8%), and trunk 14 cases (15.8%). Tumor staging was made according to the Enneking staging system and the American Joint Committee on Cancer (AJCC) 7th edition staging system. Of the 89 patients, 41 were in stage IIA (46.1%) and 48 in stage IIB (53.8%) as per the Enneking staging system, but as per the AJCC staging system, 35 patients were in stage II (39.3%) and 54 patients in stage III (60.7%).

Furthermore, there were 75 patients receiving neoadjuvant chemotherapy (84.3%), 9 patients receiving adjuvant chemotherapy (10.1%), and 5 patients not receiving chemotherapy (5.6%). Of the 84 patients receiving chemotherapy, 39 received less than six treatment courses and 45 received more than six treatment courses.

In the first treatment of the 89 patients, 81 (91%) received extended resection in the tumor site and 8 received amputation rather than limb-sparing surgery due to neoplasm invading important nerves and blood vessels (Table 2).

**Table 2:** Baseline information of patients

Characteristic	Patients	Neoadjuvant chemotherapy	Non-neoadjuvant chemotherapy
Number	89	75 (84.3%)	14 (15.7%)
Gender			
Male	46	40 (87%)	6 (13%)
Female	43	35 (81.4%)	8 (18.6%)
Age			
<25 years old	30	25 (83.3%)	5 (16.7%)
≥25 years old	59	50 (84.7%)	9 (15.3%)
Tumor size			
≤5 cm	35	30 (85.7%)	5 (14.3%)
>5 cm	54	45 (83.3%)	9 (16.7%)
Primary tumor site			
Limb			
Upper limb	23	20 (87%)	3 (13%)
Lower limbs	52	43 (82.7%)	9 (17.3%)
Trunk	14	12 (85.7%)	2 (14.3%)
Shoulder-back	8	6 (75.0%)	2 (25.0%)
Hip	2	2 (100%)	0
Chest wall	2	2 (100%)	0
Pelvis	1	1 (100%)	0
Neck	1	1 (100%)	0
Pathological pattern			
Unipolar type	41	31 (75.6%)	10 (24.4%)
Bipolar type	28	26 (92.8%)	2 (7.2%)
Undifferentiated type	8	8 (100%)	0 (0%)
Unknown	12	10 (83.3%)	2 (16.7%)
Enneking staging			
Phase IIA	41	33 (80.4%)	8 (19.6%)
Phase IIB	48	42 (87.5%)	6 (12.5%)
AJCC staging			
Phase II	35	30 (85.7%)	5 (14.3%)
Phase III	54	45 (83.3%)	9 (16.7%)
Chemotherapy course			
<6	39		
≥6	45		
No	5		
Surgical method			
Amputation	8	8 (100%)	0 (0%)
Extended resection	81	67 (%)	14 (%)
Surgical margin			
Positive	3	3 (100%)	0 (0%)
Negative	86	72 (83.7%)	14 (16.3%)
The 1st visiting hospital			
Our hospital	20	17 (85%)	3 (15%)
Other hospital	69	58 (84.1%)	11 (15.9%)
Local recurrence			
Yes	20	17 (85%)	3 (15%)
No	69	58 (84.1%)	11 (15.9%)
Distant metastasis			
Yes	17	12 (70.6%)	5 (29.4%)
No	72	63 (87.5%)	9 (12.5%)
Last follow-up state			
Died from disease	16	11 (68.7%)	5 (31.3%)
Died from other reasons	1	0 (0%)	1 (100%)
Survived	72	64 (88.8%)	8 (11.2%)

### Treatment outcome

**Fig. 1:**
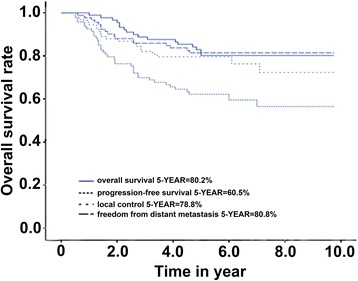
Overall survival (OS), progression-free survival (PFS), local control, and freedom from distant metastasis (FFDM)

The median follow-up time was 68.6 months (12~117 months), and that of the patients who survived was 76.3 months (52~117 months). Furthermore, at the last follow-up of the 89 patients, 72 survived and 17 died. Among the 17 patients who died, 16 died from tumor pulmonary metastasis and 1 died from other reasons. Until the last follow-up, 20 patients showed local recurrence, among whom 18 underwent extended resection in the tumor site and 2 amputation. In addition, 1 of the 20 patients who showed local recurrence died from pulmonary metastasis, and the others survived. Until the last follow-up, 17 patients showed distant metastasis, 16 of whom died from pulmonary metastasis and the remaining 1, who showed lymphatic metastasis, survived after extended resection (Table 2). The 5-year OS of the 89 patients was 80.2%, and the 5-year PFS was 60.5%. The 5-year LC and 5-year FFDM were 78.8 and 80.8%, respectively (Fig. 1).

### Influencing factor for prognosis

**Fig. 2:**
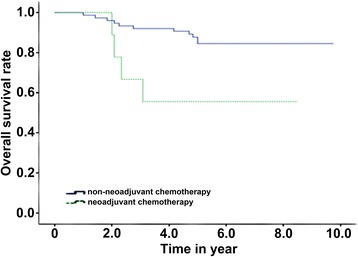
Total overall survival (OS) for different chemotherapy methods

**Fig. 3:**
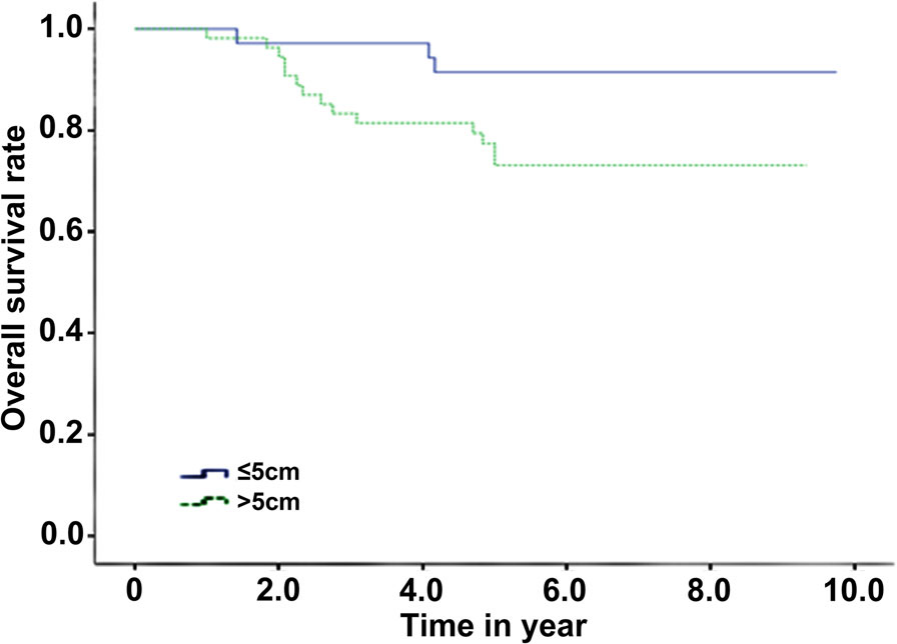
Total overall survival (OS) for different tumor sizes

**Fig. 4:**
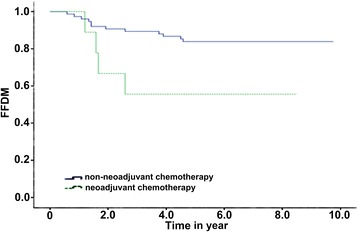
Freedom from distant metastasis (FFDM) for different chemotherapy methods

Univariate analysis and multivariate analysis showed that the chemotherapy method and tumor size were the independent factors influencing the patients’ OS (*P* < 0.05). Furthermore, the chemotherapy method was also the independent factor influencing FFDM (*P* < 0.05) as shown in Table 3. The OS and FFDM of neoadjuvant chemotherapy were better than those of adjuvant chemotherapy (*P* = 0.028). The 5-year OS was 84.5% for the patients receiving neoadjuvant chemotherapy and was 55.6% for the patients receiving adjuvant chemotherapy (Fig. 2). The tumor size could influence the patients’ OS, and the OS was lower when the size was larger than 5 cm (*P* < 0.05). The 5-year OS of the patients with a tumor size ≤5 cm was 91.4%, and that of the patients with a tumor size >5 cm was only 73.1% (Fig. 3). The 5-year FFDM of the patients receiving neoadjuvant chemotherapy was 83.8%, and that of the patients receiving non-neoadjuvant chemotherapy was 55.6% (Fig. 4). In this study, age (<25 or ≥25), chemotherapy treatment course (<6 or ≥6), and tumor site (extremities or trunk) had no significant influence on OS, PFS, LC, and FFDM.

**Table 3:** Results of single-factor analysis

Influencing factor	Number	5-year OS (%)	*P*	5-year PFS (%)	*P*	5-year LC (%)	*P*	5-year FFDM (%)	*P*
Totality	89	80.2		60.5		78.8		80.8	
Age			0.302		0.501		0.979		0.685
<25 years old	30	86.1		66.5		79.7		83.2	
≥25 years old	59	77.1		57.5		78.4		79.5	
Tumor size			0.048		0.606		59.2		0.053
≤5 cm	35	91.4		65.7		76.8		91.4	
>5 cm	54	73.1		57.2		80.0		73.9	
Chemotherapy method			0.015		0.313		0.883		0.028
Adjuvant chemotherapy	9	55.6		44.4		71.1		55.6	
Neoadjuvant chemotherapy	75	84.5		63.8		79.3		83.8	
Chemotherapy course			0.287		0.726		0.958		0.403
<6	39	76.8		59.0		78.7		76.9	
≥6	45	85.2		64.0		79.0		84.1

## Discussion

Surgery is the most common treatment for synovial sarcoma, resulting in a good prognosis. Till date, there is minimal information available on surgery-based combined treatment modalities and there is lack of data to prove their statistical significance over surgery. So to better elucidate whether clinicopathological characteristics and treatment are correlated with survival in patients with synovial sarcoma and to find specific prognostic factors, this study has been designed. The major results of this study are that, in the 89 patients with synovial sarcoma, the 5-year OS was 80.2% and neoadjuvant chemotherapy and tumor size were independent factors influencing the patients’ survival time. Furthermore, neoadjuvant chemotherapy was also an independent factor influencing FFDM, suggesting that the application of neoadjuvant chemotherapy is of great clinical significance for improving synovial sarcoma patients’ prognosis.

In reports during the 1990s, when synovial sarcoma was considered as a high-level malignant sarcoma, the 5-year OS of patients with the disease was 40~76% [8–11]. Recently, there were many reports on synovial sarcoma patients without metastasis in the primary diagnosis. Shi et al. [12] reported that in a follow-up study of 92 patients in a single-center study, the 5-year OS was 61%; Ferrari et al. reported 64.3% (271 patients) [5]; Deshmukh et al. reported 70% (108 patients) [13]; Guadagnolo et al. reported 76% (150 patients) [6]; Palmerini et al. reported 76% (204 patients) [14]. Here, the 5-year OS of patients with synovial sarcoma was 80.2%, similar to those of the recent studies but with slight improvement. The higher 5-year OS of patients with synovial sarcoma might be related to the application of neoadjuvant chemotherapy to the patients enrolled in this study (84.3%).

Local extended resection combined with radiotherapy has been widely applied in patients with synovial sarcoma overseas, reducing the local recurrence rate and increasing the OS [6, 12, 15]. In addition, radiotherapy was not widely used in treating patients in early years, but a satisfactory result was also achieved by using local extended resection and neoadjuvant/adjuvant chemotherapy. In addition, synovial sarcoma was considered as a chemo-sensitive tumor [16–18]. Eilber et al. [19] reported that the 4-year OS of patients receiving chemotherapy and not receiving chemotherapy was 88 and 67%, respectively, with a significant difference (*P* < 0.05) in a 101-case report. In our study, the 5-year OS of patients receiving and not receiving chemotherapy was 81.4 and 60%, respectively, which is in line with the above report.

Multicenter studies demonstrated that chemotherapy mainly on ifosfamide could improve the survival time of patients in the treatment of high-level soft tissue sarcoma [20–22], and Eilber et al. also found the same therapeutic effect in patients with synovial sarcoma. We used the MAID regimen to treat 84 patients by chemotherapy. In a previous study, Mullen et al. [23] reported that the application of neoadjuvant chemotherapy combined with radiotherapy greatly improved the OS, LC, and FFDM compared with the control group (without chemotherapy or with other chemotherapy) in treating high-level soft tissue sarcoma (*P* < 0.05). Neoadjuvant chemotherapy has been reported in the treatment of synovial sarcoma previously, but not as the main treatment. In this study, 72 patients received neoadjuvant chemotherapy and 9 received adjuvant chemotherapy; the 5-year OS was 84.5 and 55.6% (*P* < 0.05) and FFDM was 83.8 and 55.6%, respectively (*P* < 0.05). We believed that neoadjuvant chemotherapy could bring more benefits for patients with synovial sarcoma compared with adjuvant chemotherapy because it could provide more direct observation of the sensitivity of tumor to chemotherapy drugs before operation and more evidence for postoperative chemotherapy and options of chemotherapy regimens which in turn increase patients’ survival time and control distant metastasis. Mullen et al. used neoadjuvant chemotherapy to perform chemotherapy with six treatment courses [23]. Among the 84 patients in our study, there was no significant difference between the patients receiving more than six courses and those receiving less than six courses in the OS (76.8 vs 85.2%), PFS (59.0 vs 64.0%), LC (78.7 vs 79.0%), and FFDM (76.9 vs 84.1%) (*P* > 0.05). How many disease courses are appropriate for chemotherapy in patients with synovial sarcoma still needs further study.

Local recurrence of tumor is related to the tumor moderate or poor differentiation area, tumor size [24], and resection margin [6, 25–27]. The 5-year LC in our study was 78.8%, and tumor size (≤5 or >5 cm) and resection margin (positive or negative) had no significant difference in LC. Tumor size is an important factor influencing prognosis in soft connective tissue tumor [19, 27], which is also very important in synovial sarcoma [5, 12, 13, 25, 28]. A larger tumor size is a reason for poor prognosis, which was proved in our study. A tumor size >5 cm indicated poor survival time and lower FFDM. Whether age could influence the prognosis of patients with synovial sarcoma is inconclusive, and it was reported that the age grouping on the differential OS was different [5, 12, 16, 24, 29]. In our study, age had no significant difference in prognosis. It is still controversial whether unipolar type or bipolar type had influence on survival time [3, 11, 30–33], but we found that they had no significant difference in survival time.

However, there were still some limitations in this study. First, this was a retrospective study and the conclusion needs further prospective study. Secondly, the case number using neoadjuvant chemotherapy was more, but that using non-adjuvant chemotherapy was less. Therefore, a larger sample would be conducive to further verification. Because this was a single-center study with consistency in treatment and management of the patients, the conclusion is of great clinical significance for understanding the prognosis of this rare disease.

## Conclusions

Through a retrospective study of 89 patients with synovial sarcoma, it was proved that neoadjuvant chemotherapy and tumor size were the independent factors influencing patients’ survival time and neoadjuvant chemotherapy was also an independent relevant factor influencing distant metastasis. Above all, it was proved that neoadjuvant chemotherapy was conducive to patients with synovial sarcoma and could improve the survival time and control distant metastasis. This study is of great clinical significance for improving synovial sarcoma patients’ prognosis.

## Abbreviations

FFDM: Freedom from distant metastasis
LC: Local control
OS: Overall survival
PFS: Progression-free survival
